# Primary Hepatic Burkitt Lymphoma in a Kidney Transplant Recipient

**DOI:** 10.1155/2018/7425785

**Published:** 2018-05-13

**Authors:** Sophia Lionaki, Eystratios Tsakonas, Athina Androulaki, George Liapis, Panagiotis Panayiotidis, George Zavos, John N. Boletis

**Affiliations:** ^1^Department of Nephrology & Transplantation Unit, Laiko Hospital, National and Kapodistrian University of Athens, Faculty of Medicine, Athens, Greece; ^2^Department of Pathology, National and Kapodistrian University of Athens, Faculty of Medicine, Athens, Greece; ^3^Molecular Hematology Laboratory, 1st Department of Propaedeutic Medicine, National and Kapodistrian University of Athens, Faculty of Medicine, Laiko General Hospital, Athens, Greece; ^4^Transplantation Unit, Laiko Hospital, Athens, Greece

## Abstract

This is a case of a renal transplant recipient who developed a primary hepatic Burkitt lymphoma a few years after kidney transplantation. The past medical history of the patient was significant for anti-HCV positivity with liver histopathology showing minimal changes of grades 0 and 1, stage 0. She received a graft from a deceased donor, with rabbit antithymocyte globulin and methyl-prednisolone, as induction therapy, and was maintained on azathioprine, cyclosporine, and low dose methyl-prednisolone with normal renal function. Four years after KTx she presented with fatigue, hepatomegaly, and impaired liver function and the workup revealed multiple, variable-sized, low density nodules in the liver, due to diffuse monotonous infiltration of highly malignant non-Hodgkin lymphoma of B-cells, which turned out to be a Burkitt lymphoma. Bone marrow biopsy and spinal fluid exam were free of lymphoma cells. At time of lymphoma diagnosis she was shown to be positive for Epstein-Barr virus polymerase chain reaction. She received aggressive chemotherapy but died due to sepsis, as a result of toxicity of therapy.

## 1. Introduction

Burkitt lymphoma (BL) constitutes less than 1% of all non-Hodgkin lymphomas [1], and this percentage increases to 25% to 30% in patients with human immunodeficiency virus (HIV) infection. It is a highly aggressive non-Hodgkin B-cell tumour that can be classified into three variants based on clinical features and disease epidemiology [1]. Endemic BL described, introduced by pioneering studies of Dr. Dennis Burkitt, affects young children in regions of equatorial Africa and Papua New Guinea, where it accounts for around 50% of all paediatric cancers. This high incidence form of the disease classically presents as a jaw tumour and is EBV-positive in almost every case. From epidemiological studies, it is clear that endemic BL is restricted to geographical regions where* Plasmodium falciparum* malaria is holoendemic and, coincidentally, where primary EBV infection occurs at a young age. Sporadic BL occurs worldwide, has a much lower incidence, and affects children across a slightly older age range and is rarely associated with EBV except in areas like Brazil where BL incidence appears to be higher, with EBV rates sometimes exceeding 80%. The third variant (HIV-related BL) affects HIV-infected individuals. Specifically, we know that HIV infection increases BL incidence by more than 100-fold above that of the sporadic disease, and some 30–40% of these tumours are EBV-positive [1]. The tumour typically develops early in the course of HIV infection, coincident with symptoms of persistent generalized lymphadenopathy and before circulating CD4^+^ T cell numbers begin to fall. Irrespective of the subtype and the EBV status, all BL tumours are morphologically and immunophenotypically similar and share a common gene expression signature resembling that of centroblasts. The tumours are composed of a monomorphic population of rapidly proliferating medium-sized B-cells characterized by the expression of IgM, CD10, and BCL6 and bearing functionally rearranged and somatically mutated Ig sequences indicative of a germinal centroblast origin [1]. Histologically, they are largely devoid of normal infiltrating lymphocytes with the exception of scattered phagocytic macrophages, which give rise to the characteristic starry sky appearance. Another key defining feature of all three BL variants is a chromosomal translocation which juxtaposes the c-MYC oncogene on chromosome 8 and one of the three Ig loci, most frequently the Ig heavy chain locus on chromosome 14 [1]. Burkitt's lymphoma in nontransplant patients has been reportedly associated with a poor prognosis, and its diagnosis usually necessitates chemotherapy of increasing intensity according to the disease stage. The typical presentation involves rapidly growing and multifocal extranodal masses throughout the body.

We present here a case of a kidney transplant (KTx) recipient, who was diagnosed with BL a few years after KTx. The effect of chronic immunosuppression in this group of patients is the most reasonable explanation.

## 2. Clinical History

This is a case of a 51-year-old female, who was diagnosed with primary hepatic Burkitt lymphoma a few years after KTx. Her past medical history was significant for hysterectomy due to fibroids, right thyroid lobectomy for a benign nodule, and anti-HCV positivity with liver histopathology showing minimal changes of grades 0 and 1, stage 0. She received a graft from a deceased donor, with rabbit antithymocyte globulin and methyl-prednisolone, as induction therapy. She was maintained on azathioprine, cyclosporine, and low dose methyl-prednisolone with normal renal function.

**Figure 1:**
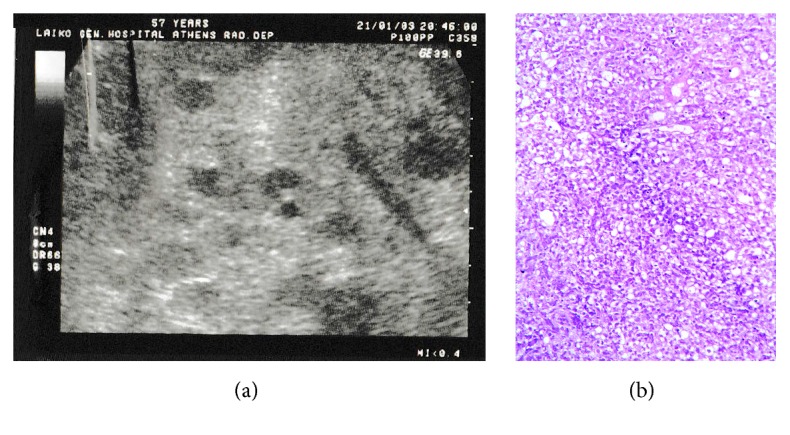
(a) Ultrasonography image showing multiple hypodense lesions suggestive of infiltration of the lymphoma. (b) Diffusely infiltrating medium-sized cells with basophilic cytoplasm, coarse chromatin, and medium-sized nucleoli are seen (H&E stain, ×400).

Four years after KTx she was admitted due to diarrhoea, vomiting, and weakness. Physical examination was remarkable for hepatomegaly with biochemistry workup showing impaired liver function. Imaging studies of the abdomen also revealed hepatomegaly (18 cm) with multiple, variable-sized, low density nodules in the liver (Figure 1(a)). Liver biopsy showed diffuse monotonous infiltration of highly malignant non-Hodgkin lymphoma of B-cells. In addition to positivity for Bcl-6 (+), CD-10 (+), and CD20 (+), L26 was also positive, indicating a BL. 100% of the cells were positive for Ki-67 (Figure 1(b)). Markers Bcl-2, TdT, and CD3 were shown negative, while phagocytosis of nuclear debris by reactive histiocytes was noted, resulting in a “starry sky” appearance.

Bone marrow biopsy and spinal fluid exam were free of lymphoma cells as was the rest of the workup, pointing to a primary hepatic BL. Maintenance immunosuppressive KTx therapy was discontinued and chemotherapy consisting of cyclophosphamide, prednisolone, methotrexate, and dexamethasone was initiated. She also received cytarabine and methotrexate intrathecally. Epstein-Barr virus polymerase chain reaction was positive and hence a therapeutic dose of acyclovir was administered. Unfortunately, chemotherapy was complicated by neutropenia and bacteraemia from* Escherichia coli* and the patient died due to sepsis despite prompt, combined treatment with advanced antibiotics and occasional administration of granulocyte-colony stimulating factor.

## 3. Discussion

Primary hepatic BL is a very rare condition with only 11 cases reported in the literature to date [2], while this is the first one to our knowledge occurring in a KTx recipient. Burkitt's lymphoma is an undifferentiated malignant lymphoma of B lymphocytes. Primary hepatic BL is defined as an extranodal lymphoma restricted to the liver, accounting for only 0.4% of all extranodal lymphomas [2, 3]. Due to rarity of its occurrence accurate epidemiology and etiology of PHBL are not established. In our patient, BL was associated with acquired immunodeficiency, due to chronic immunosuppressive therapy for KTx [4], but also with Epstein-Barr viremia [5]. Epstein-Barr virus, a member of the human herpes virus family, is a linear, double-stranded DNA virus that was initially isolated from a cultured BL cell line by Epstein et al. in 1964. Subsequent studies demonstrated that EBV was a potent growth-transforming agent for primary B-cells, and that all cases of BLs carried characteristic chromosomal translocations resulting in constitutive activation of the c-*MYC* oncogene [6]. Since the liver is a vital organ, involvement with lymphoma can lead to a rapidly progressive, life-threatening illness with high rates of mortality soon after presentation [5]. Besides, BL is an aggressive mature B-cell non-Hodgkin lymphoma, which follows a rapid clinical course and can be fatal soon after presentation if left untreated. Early assessment of the correct diagnosis is crucial for disease management. Primary hepatic lymphoma, although relatively rare, should be considered as one of the differential diagnosis of liver lesions especially in patients who are chronically immunosuppressed.

Although malignancies justify aggressive therapies, in some settings it may be difficult for the patient, especially if they are already immunocompromised, to tolerate it without significant adverse events and/or infective episodes. In this regard, our patient received a scheme, highly effective for such circumstances. However, she developed an infection, which became fatal, despite full treatment with appropriate antibacterials and granulocyte-colony stimulating factor, probably because she had already accumulated a significant burden of immunosuppression from the years of KTx and thus she entered a state of imbalance between toxicity and efficacy soon after the initiation of therapy for BL.
